# Characteristics of postoperative early and late period for surgical treatment of true aneurysms of subclavian and axillary arteries

**DOI:** 10.1186/1749-8090-8-S1-P45

**Published:** 2013-09-11

**Authors:** O Gokalp, I Yurekli, L Yilik, U Yetkin, T Gunes, M Akyuz, B Ozcem, O Tetik, G Ilhan, A Gurbuz

**Affiliations:** 1 Izmir Katip Celebi University Ataturk Training and Research Hospital, Department of Cardiovascular Surgery, Turkey

## Background

Both subclavian and axillary artery aneurysms should be promptly treated after diagnosis due to complications. The primary treatment modality is mainly surgery.

## Methods

Eight patients that were operated on between February 1998 and December 2007 due to true aneurysms of the subclavian and axillary arteries were examined retrospectively in terms of clinical and perioperative parameters. Median age of the patients was 58 (38-73). Six of them were male (75%). Six patients had axillary and 2 patients had subclavian artery aneurysms. : Postoperative intubation time, duration of intensive care unit stay, amount of blood products used, complications as surgical wound infections and bleeding were recorded. After the discharge, patients were examined with Doppler ultrasound at the 1st month and 1st year control visits.

## Results

Duration of the operation was 2 hours (1-3). Median intubation period was 3.5 hours (2-5). Median duration of intensive care unit stay was 1.5 days (1-2). Median duration of hospital stay was 3 days (2-4). Median unit of blood products used perioperatively was 1 unit (0-3). As early postoperative complication, one patient developed perianastomotic pseudoaneurysm 40 days after the initial operation due to subclavian artery aneurysm. This patient underwent subclavian-axillary bypass using saphenous vein in the second operation again. Another patient that underwent emergency operation due to bleeding had weakness of the affected upper extremity due to preoperative brachial plexus injury. But in order to disappear completely, he needed physical therapy for some period. No early postoperative mortality was seen. Only one patient died 5 years after the operation due to some unrelated causes. Median follow-up period was 106 months (64-170). Secondary patency rate was 100% during follow-up.

## Conclusion

True aneurysms of the subclavian and axillary arteries are rarely seen. After the diagnosis, the successful surgical repair is possible.

